# Accuracy of the ACS-NSQIP Risk Calculator, Nottingham Hip Fracture Score and CRP/Albumin Ratio in Predicting Adverse Postoperative Outcomes in the Hip Fracture Surgery: A Lithuanian Single-Centre Prospective Study

**DOI:** 10.1177/21514593251352336

**Published:** 2025-06-25

**Authors:** Povilas Masionis, Rokas Bobina, Simonas Utkus, Raminta Martinaitytė, Valentinas Uvarovas, Igoris Šatkauskas

**Affiliations:** 1 Clinic of Rheumatology, Orthopaedic Traumatology and Reconstructive Surgery, Institute of Clinical Medicine, Faculty of Medicine, Vilnius University; Centre of Orthopedics and Traumatology, Vilnius Republican University Hospital, Vilnius, Lithuania

**Keywords:** hip fracture, hemiarthroplasty, total hip arthroplasty, osteosynthesis, mortality

## Abstract

**Objective:** As the global population ages, hip fracture importance will increase. The high postoperative mortality and morbidity necessitate tools for accurate risk assessment to aid surgical decisions and inform patients and families. This study aimed to compare and validate ACS NSQIP, Nottingham hip fracture risk calculators, and C reactive protein/albumin ratio in predicting complications and 30 day mortality.

**Methods:** 583 patients over the 65 years old who sustained hip fracture from simple fall and underwent surgical treatment for hip fracture were included in prospective study. Each patient was evaluated by ACS NSQIP and Nottingham hip fracture risk calculators and C reactive protein/albumin ratio was calculated from preoperative values. Patients were followed up for 30 days and all the complications were recorded.

**Results:** ACS NSQIP and Nottingham hip fracture score showed AUC of .724 and .731 respectively. C reactive protein/albumin ratio performed less and showed AUC of .623 that is defined as poor predictor for 30 day mortality. Furthermore, in terms of predicting any complication, ACS NSQIP showed AUC of .645, Nottingham hip fracture score of .611 and C reactive protein/albumin ratio of .594. Nottingham hip fracture score than compared to ACS NSQIP yielded lower average of mortality rates (5.4% compared to 7.9%) in contrast of study findings of 8.1%. None of the ACS NSQIP scale predictive complication showed acceptable performance. When adjusted for fracture type, Nottingham hip fracture score showed .858 AUC in predicting 30 day mortality in femoral neck fractures.

**Conclusions:** We recommend Nottingham fracture risk calculator use for 30 day mortality risk assessment in femoral neck fractures. In hip fractures combined—none of models showed strong discrimination. In our cohort C reactive protein/albumin ratio showed poor prognostic values in terms of mortality and complications.

## Introduction

It is predicted that by the middle of the century, approximately 6 million people worldwide will sustain hip fractures (HF) annually.^1^ As the global population ages, the significance of this condition will continue to grow. HF patients are known to experience up to 14% early mortality and up to 40% post-operative complications.^1-6^ These poor outcomes are attributed to factors such as age, high morbidity (including hypertension, diabetes, obesity, heart failure, osteoporosis, etc.), smoking, alcohol abuse, malnutrition. Due to these reasons, HP is often regarded as “the last fracture of whole life”.^1,7-9^ Despite some studies showing a decreasing incidence in Northern countries and stabilisation in Central Europe, the rate of complications and treatment costs are expected to rise.^10^

Given the fragility of these patients, accurate preoperative assessment of mortality and morbidity risk following HF is essential to modern treatment.^5,11^ This assessment not only aids in determining the timing of surgery and providing appropriate information to the patient and their family but also identifies patients at the highest risk who require perioperative interventions.^1-14^ Understanding the risk of surgery is crucial for both the patient and the surgeon.

To address these concerns, risk calculators has been introduced. The American College of Surgeons National Surgical Quality Improvement Program (ACS NSQIP) surgical risk calculator is a unique tool designed to predict 18 different procedure-specific outcomes based on The Current Procedural Terminology (CPT) codes.^3,5,14-18^ However, its performance in HF patients is understudied. Additionally, C reactive protein/albumin ration (CAR) has previously been found to predict 30 day and 1 year mortality and morbidity in various pathologies, but its effectiveness compared to risk calculators in HF patients is unknow.^19-22^

In 2007, Nottingham hip fracture score (NHFS) was introduced and has shown reliability in predicting 30 day and 1 year mortality in HF. The NHFS was used as a control measure in the present study.^23-25^

The objective of this study was to assess and validate the ACS NSQIP, NHFS surgical risk calculators and CAR index in predicting complications and 30 day mortality among HF patients.^26^

## Methods

**Figure 1.**
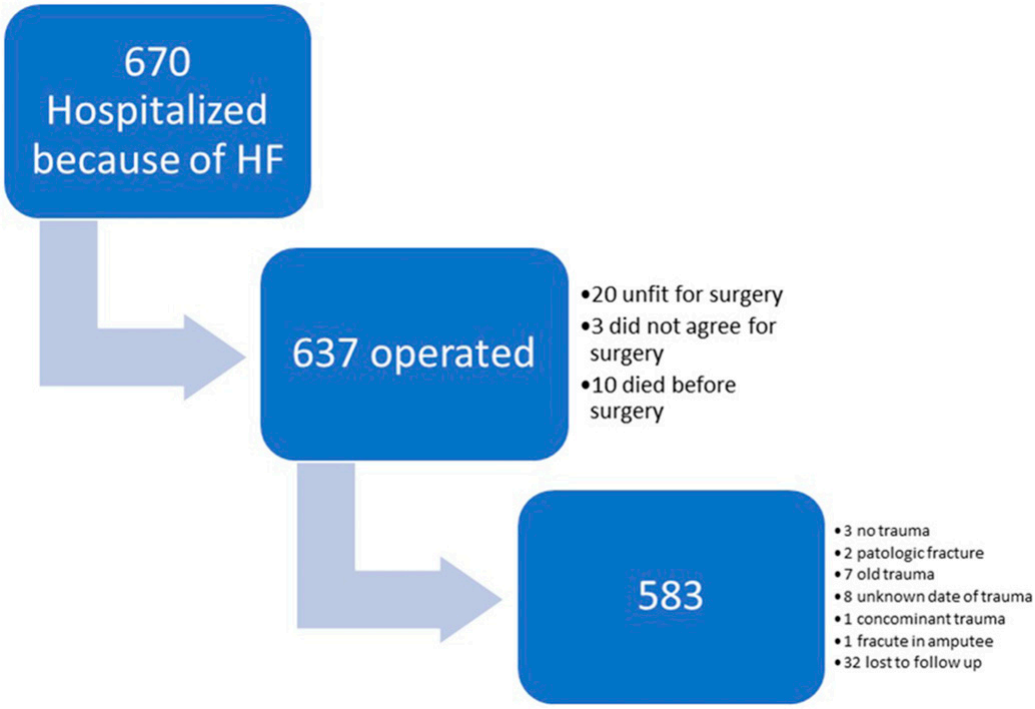
Final Sample Size of Included Patients

The manuscript was written according to STROBE guidelines. The prospective study was conducted in a tertiary orthopaedic trauma centre from December 1, 2022, to December 31, 2023. All patients or their proxies provided informed consent to participate in the study, which was approved by the Institutional Review Board of our hospital and the regional bioethics committee. The inclusion criteria were as follows: ≥65 years patients; intertrochanteric fracture; subtrochanteric fracture; femoral neck fracture; patients who underwent surgical treatment; low energy trauma (simple fall); closed fractures; isolated trauma; acute trauma. All surgical interventions were performed or supervised by a consultant orthopaedic surgeon. After surgery, patients were followed-up for 30 days. At the 30th day, patient or his proxy was contacted by the investigator for interview. Additionally, patient’s electronic medical history was examined. The following serious complications were registered: pneumonia, cardiac complications, surgical site infection, urinary tract infection, deep venous thrombosis, acute renal failure, readmission, reoperation, death, sepsis, delirium, and pressure sores. For any complication, we recorded any additional intervention or consultation that deviated from the normal treatment pathway. Furthermore, time from trauma to hospitalization, time from hospitalization to surgery, in-hospital stay, functional decline, new mobility aid, and discharge to a nursing home were recorded. General information on patients is presented in Table 1. Final sample size of 583 patients is presented in flow chart Figure 1. Upon admission, ACS NSQIP and NHFS scores were calculated. For CAR, pre-operative values of C reactive protein (CRP) and albumin were used. In our cohort we used NHFS to validate it and furthermore, as a control for ACS NSQIP and CAR performance.

**Table 1.** General information on patients.

Variables	
Gender (female/male)	452 (77.5%)/131 (22.5%)
Age	82 ± 8
Number of comorbidities	6 ± 3
Fracture type	
Neck	292 (50.1%)
Pertrochanteric	259 (44.4%)
Subtrochanteric	32 (5.5%)
Surgery type	
Hip hemiarthroplasty	191 (32.8)
Total hip arthroplasty	70 (12.0%)
Proximal femoral nail	236 (40.5%)
Dynamic hip screw	69 (11.8%)
Cannulated screws	17 (2.9%)

All analyses were conducted using IBM SPSS version 26.0 software (SPSS Inc, Chicago, IL) for Windows. Normally distributed data are presented as mean (±standard deviation). Non normally distributed data are presented as median (25-75th IQR). Shapiro-Wilk test was used to assess the normality of data distribution. Discrimination in this study was evaluated using the area under the receiver operating characteristic (ROC) curve, also known as the AUC or c-statistic. The AUC ranges from 1.0 (perfectly discriminating model) to 0.5 (no better than chance). An AUC of 0.6-0.69 indicates poor discrimination, 0.7-0.79 indicates adequate discrimination, 0.8-0.89 indicates strong discrimination, and 0.9-1.0 indicates excellent discrimination.

## Results

**Figure 2.**
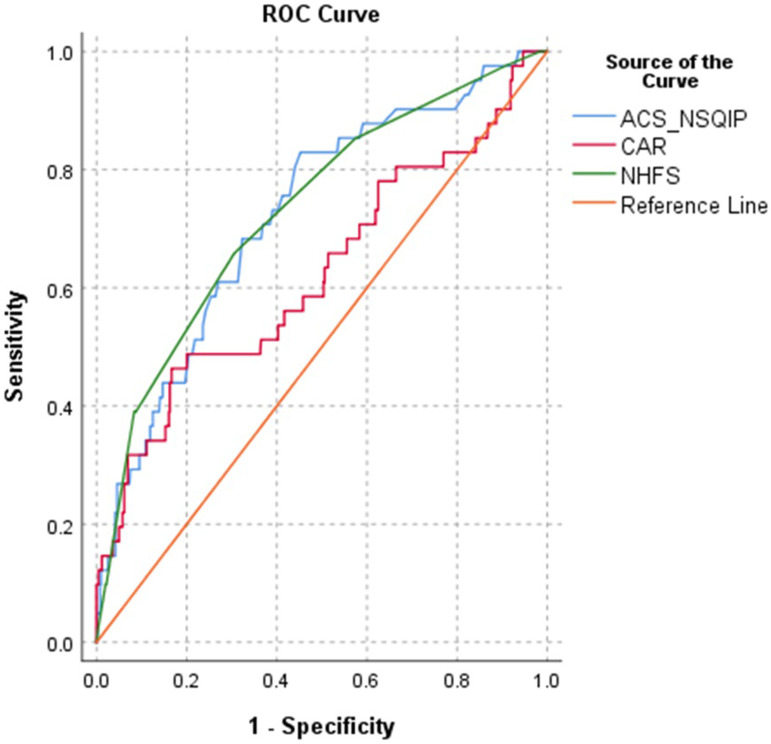
Receiver Operating Characteristics (ROC) of the The American College of Surgeons National Surgical Quality Improvement Program (ACS-NSQIP) Surgical Risk Calculator, Nottingham Hip Fracture Score (NHFS) and C Reactive Protein/Albumin Ration (CAR) for Prediction of 30 day Mortality

**Figure 3.**
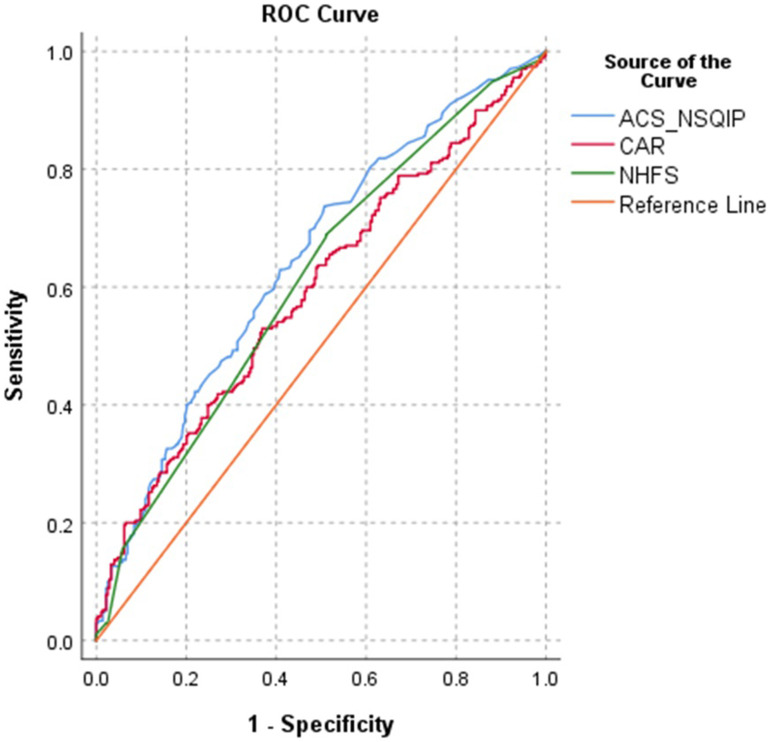
Receiver Operating Characteristics (ROC) of the The American College of Surgeons National Surgical Quality Improvement Program (ACS-NSQIP) Surgical Risk Calculator, Nottingham Hip Fracture Score (NHFS) and C Reactive Protein/Albumin Ration (CAR) for Prediction Of Any Complication

Frequencies of complications are presented in Table 2. ACS NSQIP and NHFS showed AUC of .724 and .731 respectively (Figure 2). To continue, CAR performed less and showed AUC of .623 that is defined as poor predictor for 30 day mortality (Table 3.). Furthermore, in terms of predicting any complication, ACS NSQIP showed AUC of .645, NHFS of .611 and CAR of .594—none of risk calculators showed adequate predicting power and CAR prediction of any complication was not better than chance (Figure 3). NHFS than compared to ACS NSQIP yielded lower average of mortality rates (5.4% compared to 7.9%) in contrast of study findings of 8.1%. None of the ACS NSQIP scale predictive complication showed acceptable performance, AUC values are presented in Table 4. When adjusted for fracture type, NHFS reached AUC of .858 in predicting 30 day mortality, CAR and ACS NSQIP tested for fracture type remained in the same reliability margins.

**Table 2.** Frequencies of complications.

Variables	
Serious complication	207 (35.5%)
Any complication	287 (49.2%)
Pneumonia	66 (11.3%)
Cardiac complication	68 (11.7%)
Surgical site infection	20 (3.4%)
Urinary tract infection	58 (9.9%)
Deep venous thrombosis	7 (1.2%)
Acute renal failure	26 (4.5%)
Readmission	62 (10.6%)
Reoperation	11 (1.9%)
Death	47 (8.1%)
Discharge to nursery	269 (46.1%)
Sepsis	10 (1.7%)
Delirium	59 (10.1%)
Functional decline	406 (69.6%)
New mobility aid	556 (95.4%)
Pressure sores	51 (8.7%)

**Table 3.** AUC and p values of risk calculators performances in prediction of death and any complication.

	ACS NSQIP	NHFS	CAR
Death	.724 (.642-.805) *P* < .001	.731 (.648-.815) *P* < .001	.623 (.521-.725) *P* = 018
Any complication	.645 (.599-.691) *P* < .001	.611 (.564-.658) *P* < .001	.594 (.547-.642) *P* <001

**Table 4.** AUC and p values of ACS NSQIP risk calculator performance in predicting complications.

	ACS NSQIP AUC (CI) and *P* value
Pneumonia	.688 (.619-.757) *P* < 000
Cardiac complications	.669 (.603-.735) *P* < 000
Surgical site infection	.628 (.482-.773) *P* = .087
Urinary tract infection	.571 (.487-.655) *P* = .097
Deep venous thrombosis	.473 (.226-.720) *P* = .830
Acute renal failure	.668 (.543-.794) *P* = .008
Readmission	.497 (.418-.575) *P* = 933
Reoperation	.643 (.498-.787) *P* = .053
Discharge to nursery hospital	.467 (.420-.514) *P* = .172
Sepsis	.643 (.465-.822) *P* = .114
Delirium	.655 (.578-.732) *P* < .000
Pressure sores	.523 (.474-.571) *P* = .356
New mobility aid	.598 (.509-.687) *P* = .030
Functional decline	.625 (.550-.700) *P* = .001

## Discussion

Surgical risk calculators are highly valuable tools that enhance the understanding of patients and their families, as well as assist in making surgical decisions based on numerical data. In our cohort, none of the models demonstrated excellent discrimination results (AUC >.80) in predicting 30 day mortality and morbidity among HP patients. Since the AUC is greater than 0.7, both the ACS NSQIP and NHFS are effective predictors of 30 day mortality. Only after adjusting for fracture type, did the NHFS show excellent discrimination for 30 day mortality in patients with femoral neck fractures. It should also be noted that the NHFS predicted a lower mortality rate than what was observed in the current study.

To our knowledge, this is the first study to compare CAR to risk calculators in the same cohort. CAR was ineffective in predicting early mortality and even worse in predicting morbidity in our study. This result contrasts with findings in the literature: Cacciola et al found CAR .816 as a reliable predictor, and Balta et al reported an AUC of .727 for intertrochanteric fractures.^22,27^ It is important to note that both previous studies were retrospective, and at the time of writing, we did not identify any prospective study ROC analysis of CAR in HF patients. Additionally, the timing of CRP sample collection might be crucial—we only included acute fractures, and in this scenario, CRP may not be elevated. However, this hypothesis needs further investigation. Although CAR has been identified as a valuable predictor in various pathologies, our results indicate that its capabilities are understudied in HF patients.

ACS NSQIP is a recent tool that utilizes procedure-specific prediction based on CPT code. The calculator has been shown to be a reliable predictor in various fields of elective and emergency settings. ROC analysis indicates it is adequate predictor of 30 day mortality; however, its ability to predict specific complications is limited, which is a notable drawback in our settings. Additionally, ACS NSQIP was developed using patient data exclusively from United States NSQIP hospitals, which may contribute to its poor discrimination in other countries.^3^ Nonetheless, this tool is continuously updated and should be evaluated over time as its design shows significant potential.

The ACS NSQIP calculator was developed from the National Surgical Quality Improvement Program (NSQIP) data to enhance surgical care quality in the USA. Harris et al^11^ later created the Hip Fracture Surgery Risk Calculator using the same data, available at https://s-spire-clintools.shinyapps.io/hip_deploy/. The model predicts 30-day mortality with an AUC of .76. Depending on the mortality risk threshold, its sensitivity and specificity vary: a 15% threshold shows 97% specificity and 78% sensitivity. These results suggest that while the model is predictive, it may overestimate mortality for some patients who could benefit from surgery. Surgeons should consider this when making treatment decisions with patients. We did not analyse this model as it was unavailable during development of our study. The author also suggested analysing the performance of risk calculators on patients who were not operated, which has not been done to our knowledge in literature and is 1 of the limitations of our study.

Originally, NHFS was developed to assess the risk for all types of proximal femur fractures. However, intracapsular fractures have been found to have higher mortality and morbidity than compared to extracapsular ones.^23-25^ This suggests that NHFS could be used for mortality risk stratification for femoral neck fractures in our settings. Moreover, NHFS is a reliable predictor and easy to use, as it only requires preoperative data. To continue, as NHFS inputs objective values, it should be less vulnerable to cultural and social differences among different populations. However, external validation in each country should be conducted before its use.

The observed complication rate in our cohort was 49.2%, which aligns with the 12.5–75% range reported in the literature.^2^ The variation in complication rates found in the literature is largely due to differences in study methodologies—retrospective studies typically report lower complication rates compared to prospective studies.^2,28^ Additionally, follow-up times vary across the literature and should be considered. Furthermore, complications are documented differently across studies. In our study, we registered serious complications according to the ACS NSQIP questionnaire, and for any complication, we recorded every event requiring additional intervention or consultation that deviated from the normal treatment pathway. This methodology for documenting all complications may have increased our reported complication rate. Generally, HF patients should be expected to experience complications, and they and their relatives must be properly informed about this possibility. Considering that NHFS uses fewer preoperative values, it is easier to use compared to ACS NSQIP. In our cohort, NHFS demonstrated strong discrimination in predicting 30 day mortality in intracapsular fractures. Based on these findings, NHFS is recommended for risk stratification in femoral neck fractures within our settings. Additionally, the results indicate that intracapsular and extracapsular proximal femur fractures, which require different surgical approaches, might benefit from fracture-specific risk calculators.

As with all studies, this study has limitations. Firstly, it is a single-centre study of 1 hospital cohort, we cannot confirm that the same results would apply to the entire population. Secondly, we only included risk calculators specific to hip fractures and excluded tools popular in other surgical fields (eg, P-POSSUM, E-PASS, Clarkson comorbidity index). These calculators were excluded purely for methodological reasons—expanding study requires additional human resources and working time. Additionally, recent tools such as the Hip Fracture Surgery Risk Calculator were not available at the time of the study but should be considered for comparison in future research.^2^ Lastly, the tools should be evaluated for their sensitivity and specificity.
